# Flame Retardancy of Sorbitol Based Bioepoxy via Combined Solid and Gas Phase Action

**DOI:** 10.3390/polym8090322

**Published:** 2016-08-30

**Authors:** Beáta Szolnoki, Katalin Bocz, György Marosi, Andrea Toldy

**Affiliations:** 1 Department of Organic Chemistry and Technology, Faculty of Chemical Technology and Biotechnology, Budapest University of Technology and Economics, Budafoki út 8, H-1111 Budapest, Hungary; bszolnoki@mail.bme.hu (B.S.); kbocz@mail.bme.hu (K.B.); gmarosi@mail.bme.hu (G.M.); 2 Department of Polymer Engineering, Faculty of Mechanical Engineering, Budapest University of Technology and Economics, Műegyetem rkp. 3, H-1111 Budapest, Hungary

**Keywords:** bioepoxy, phosphorous additive FR, solid and gas phase mechanism, laser pyrolysis-FTIR coupled method, TGA

## Abstract

Flame-retarded bioepoxy resins were prepared with the application of commercially available sorbitol polyglycidyl ether (SPE). The additive-type flame retardancy of the cycloaliphatic amine-cured SPE was investigated. Three-percent phosphorus (P)-containing samples were prepared with the application of the liquid resorcinol bis(diphenyl phosphate) (RDP), the solid ammonium polyphosphate (APP), and by combining them. Synergistic effect was found between the inorganic APP and the organophosphorus RDP, when applied in combination: formulations applying RDP or APP alone showed increased limiting oxygen index (LOI) values, however, their UL-94 standard ratings remained HB. When the same amount of P originated from the two additives, V-0, self-extinguishing rating and LOI value of 34% (*v/v*) was reached. By the combined approach the heat release rate of SPE could be lowered by approximately 60%. The assumed balanced solid and gas phase mechanism was confirmed by thermogravimetric analysis, Fourier transform infrared spectrometry (FTIR) analysis (of the gases formed during laser pyrolysis), attenuated total reflection-infrared spectrometry (ATR-IR) analysis (of the charred residues), as well as by mechanical testing (of the char obtained after combustion).

## 1. Introduction

Replacement of metallic structures by epoxy resin (EP) composites of high mechanical performance is rapidly increasing in many industrial segments; however, compared to metals, the flammability of their organic matrix represents a major disadvantage. The need for effective flame retardancy solutions is even more emerging with the spread of renewable resins and their composites reinforced with natural fibres [1,2]. Cured epoxy resins, and especially the most sugar-based bioepoxy resins have a high concentration of OH-groups [3,4] and, therefore, phosphorus-containing fire retardants are particularly effective in their case. The flame retardant efficacy of the different phosphorus-derivatives, which can be applied both as additives and reactive components is well-known. They can act both in gas and solid phase: In the gas phase, the formed HPO· and PO· radicals can react with the H· and OH· radicals, reducing the reaction rate of the radicals in the flame [5,6]. In the solid phase, phosphorous compounds promote the charring of the surface, thus, the formed thermal insulating layer can protect the material from further degradation [7].

Among the additive type phosphorus flame retardants (FRs) ammonium polyphosphate (APP) is one of the most widely used additives, which acts in the solid phase [8]. In the case of epoxy resins mainly the fire retardancy of diglycidyl ether of bisphenol A (DGEBA) was examined with APP. By using low molecular mass polyamide as hardener already the application of 5% APP was sufficient to reach V-0 UL-94 classification [9] while, in the case of cycloaliphatic amine crosslinking agent, 9% of microencapsulated APP (equivalent to approximately 3% phosphorus (P)) was needed to reach the same result [10]. As epoxy resins are mainly liquid, the use of liquid flame retardant additives are preferred in many applications, in order to avoid homogenization problems, aggregation, and/or filtration of solid particles e.g., in case of composite preparation by resin transfer moulding. A promising candidate among liquid ones is resorcinol bis(diphenyl phosphate) (RDP), having high thermal stability and low volatility [11]. Its fire retardant effect has been mainly studied in polycarbonate and acrylonitrile butadiene styrene copolymer [12,13,14,15], and it has been concluded that RDP mainly acts through flame inhibition in the gas phase. In the case of epoxy resin toughened with polyethersulfone, the limiting oxygen index (LOI) of the system could be improved from 24% to 34% (*v/v*) by adding 8% RDP (equivalent to 0.86% P) [16].

Although it is well-known, that it is advantageous, if the applied flame retardant or flame retardant combination performs action in both gas and solid phase [17], and it is suspected that the flame retardancy synergism of combining less volatile and more volatile phosphate flame retardants can be attributed to a combined phase action [8,18], to best of our knowledge no systematic study was carried out on the flame retardancy mechanism of combinations of solid and gas phase flame retardants.

In this work fire retardancy of commercially available sorbitol polyglycidyl ether (SPE) was investigated using APP, acting in solid phase, RDP, acting mainly in gas phase, and their combination. The change of the glass transition temperature, due to their effect, was determined by differential scanning calorimetry, while their fire retardancy was evaluated by limiting oxygen index (LOI), UL-94 tests and mass loss calorimetry. The anticipated combined solid and gas phase mechanism was confirmed by thermogravimetric analysis, Fourier transform infrared spectrometry (FTIR) analysis of the gases formed during laser pyrolysis, attenuated total reflection-infrared spectrometry (ATR-IR) analysis of the charred residues, as well as by mechanical resistance testing of the chars obtained after combustion, carried out by a plate-plate rheometer.

## 2. Materials and Methods

### 2.1. Materials

A commercially available renewable sugar-based component, sorbitol polyglycidyl ether (SPE) was used as the epoxy (EP) monomer (Emerald Performance Materials (Moorestown, NJ, USA), trade name: ERYSIS GE-60, epoxy equivalent weight: 160–190 g/mol, viscosity at 25 °C: 7–9.5 Pa·s, density at 25 °C: 1.27–1.30 g/cm^3^).

Cycloaliphatic amine, MH 3122 (formerly known as T-58) was the hardener (Ipox Chemicals (Budapest, Hungary) main component: 3,3’-dimethyl-4,4’-diaminodicyclohexylmethane, amine hydrogen equivalent: 60 g/equivalent, viscosity at 25 °C: 80–120 mPa∙s, density at 25 °C: 0.944 g/cm^3^).

The SPE matrix consisted of 75 wt % EP monomer and 25 wt % hardener.

As flame retardant (FR) additives resorcinol bis(diphenyl phosphate) (RDP) (ICL Industrial Products (Beer Sheva, Israel), trade name: Fyrolflex RDP, P-content: 10.7%) and ammonium polyphosphate (APP) (Nordmann Rassmann (Hamburg, Germany), trade name: NORD-MIN JLS APP, P-content: 31%–32%, average particle size of 15 μm) were applied.

**Figure 1:**
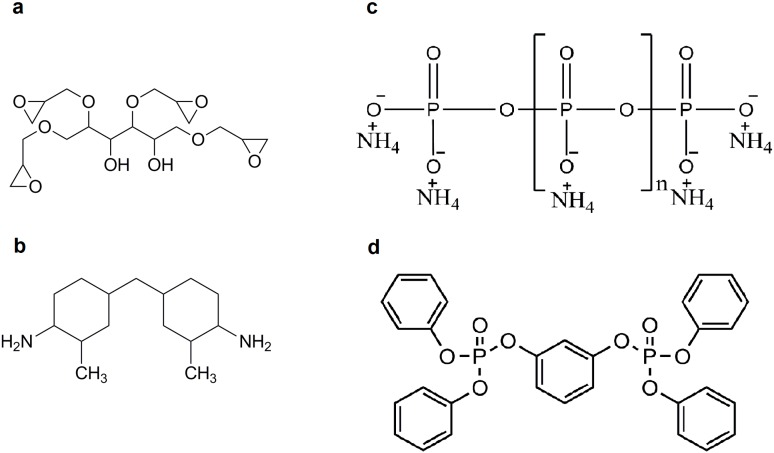
Chemical structures of the applied components sorbitol polyglycidyl ether (SPE) (**a**); Cycloaliphatic amine (T-58) (**b**); ammonium polyphosphate (APP) (**c**); and resorcinol bis(diphenyl phosphate) (RDP) (**d**).

The chemical structures of the applied EP monomers and FR additives can be seen in Figure 1.

### 2.2. Methods

#### 2.2.1. Matrix Sample Preparation

During the specimen preparation in all cases stoichiometric ratio of the EP component and hardener was used. Epoxy resin samples of gradually increasing phosphorus content (1%, 2%, and 3%) were prepared. First, the flame retardant (FR) component (APP, RDP, or both) was mixed with the SPE epoxy component. Then the curing agent was added and mixed at room temperature in a crystallizing dish in order to obtain a homogenous mixture. The samples were cured in appropriately-sized silicon moulds. The curing procedure, determined on the basis of differential scanning calorimetry (DSC) and gel time tests, consisted of the following isothermal heat steps: 2 h at 80 °C, 2 h at 120 °C.

#### 2.2.2. Differential Scanning Calorimetry (DSC)

The DSC tests were carried out with Q2000 device of TA Instruments (New Castle, DE, USA) in 50 mL/min nitrogen flow. Tzero-type aluminium pans were used, the sample mass was 5–10 mg. For the investigation of the curing process of the samples the applied three-step temperature program consisted of heat/cool/heat cycles: after a linear ramp from 25–250 °C with 5 °C/min heat rate (first cycle), the sample was cooled down to 0 °C with 50 °C/min cooling rate, followed by a second linear heating ramp from 0–250 °C with 5 °C/min heating rate (second cycle) to ensure the proper conversion. The glass transition temperature (*T*_g_) values were determined from the second heating scan and were defined as the inflection point of the transition curve.

#### 2.2.3. Characterization of the Fire Behaviour

The fire behaviour of the reference and flame retarded systems was characterized by limiting oxygen index measurements (LOI, according to ASTM (American Society for Testing and Materials) D2863 standard). The LOI value expresses the lowest volume fraction of oxygen in a mixture of oxygen and nitrogen that supports flaming combustion of a material under specified test conditions. The sample size was 120 mm × 15 mm × 4 mm.

UL-94 flammability tests (according to Standard for Tests for Flammability of Plastic Materials for Parts in Devices and Appliances, ASTM D3081 and ASTM D635, respectively) were also carried out in order to classify the samples based on their flammability in horizontal and vertical test setups. The sample size was 120 mm × 15 mm × 4 mm. The increasing values of UL-94 ratings are as follows: HB, V-2, V-1, V-0.

Mass loss type cone calorimeter tests were carried out by an instrument made by FTT Inc. (East Grinstead, UK) using the ISO 13927 standard method. Specimens (100 mm × 100 mm × 2 mm) were exposed to a constant heat flux of 25 kW/m^2^ and ignited. Heat release values and mass reduction were continuously recorded during burning. The heat insulation properties of the developed chars were investigated during the mass loss type cone calorimeter test: three thermometers were placed under the aluminium sample holder, and the temperature was continuously measured.

#### 2.2.4. Thermogravimetric Analysis (TGA)

The thermal stability of the prepared FR formulations was determined using a TA Q5000 device of TA Instruments (New Castle, DE, USA) in the range of 25–800 °C, with a heating rate of 10 °C/min, under nitrogen gas flow rate of 30 mL/min. Platinum-HT type sample pan was used, the sample size was about 15 mg in each case.

#### 2.2.5. Laser Pyrolysis–Fourier Transform Infrared Analysis

The laser pyrolysis–Fourier transform infrared (LP-FTIR) [19] method was used for investigating the pyrolytic degradation products of samples, and so the possible gas-phase effect of the different flame retardants. The system comprises of a CO_2_ pyrolyser laser (10.6 nm, SYNRAD 48-1, Mukilteo, WA, USA) unit coupled with Bruker Tensor 37 type FTIR spectrometer (Billerica, MA, USA) (detector: deuterated triglycine sulfate (DTGS), gas cell: KRS5 type thallium bromo-iodide window, resolution: 4 cm^−1^). The pyrolysis of the samples was carried out with 1 W laser power for 1 min, and the formed gases were subjected to FTIR analysis.

#### 2.2.6. Attenuated Total Reflection Infrared (ATR-IR) Analysis of the Charred Residues

IR spectra of the charred residues received after mass loss type cone calorimeter tests were recorded in ATR mode in wavenumber region of 4000–600 cm^−1^, using the same Bruker Tensor 37 FTIR spectrometer.

#### 2.2.7. Char Strength Determination

The mechanical resistance of the chars obtained after combustion of a round specimen (with a diameter of 25 mm and a thickness of 2 mm) in the mass loss type cone calorimeter (set to 50 kW/m^2^ heat flux) was examined through compression tests carried out in a TA AR2000 Rheometer (New Castle, DE, USA) with plate-plate geometry, with a constant squeeze rate of 30 μm/s. During the test the normal force transduced by the charred layer was constantly detected and registered [20,21,22,23].

## 3. Results

### 3.1. Glass Transition Temperature

Generally, the addition of flame retardants significantly influences the glass transition temperature (*T*_g_) of the matrix polymer, and consequently its applicability as well. The *T*_g_ of the flame retarded SPE samples determined by DSC can be seen in Table 1.

The plasticizing effect of the additives becomes more pronounced in the case of liquid RDP: by increasing its amount, the *T*_g_ is gradually decreasing. By adding 3% P from RDP the decrease in *T*_g_ is 29 °C. In case of APP, due to its higher P-content, a lesser amount is needed to reach the same P-content of the sample than in case of RDP. Furthermore, well-dispersed rigid APP particles can block the segmental movements in the cross-linked epoxy matrix and can compensate the decrease of *T*_g_ caused by the reduced degree of crosslinking in the presence of filler particles [24]. Upon increasing its ratio in the polymer, the *T*_g_ remained uniformly 110 °C, independently from the APP concentration. Most probably at higher APP loadings the dispersion is less efficient; therefore, no increase in *T*_g_ was detected by increasing the APP ratio. In mixed FR formulations the *T*_g_ decrease was even less than in the case of APP, independently from the origin of their P-content, the *T*_g_ decreased by 10 °C. Comparing the RDP (1% P) + APP (2% P) sample with the RDP (1% P) sample, it can be concluded the addition of 2% P from APP to 1% P from RDP, did not result in further decrease in *T*_g_, both samples have a *T*_g_ of 114 °C. By increasing the ratio of RDP and decreasing the ratio of APP, the *T*_g_ remained 114 °C, which can be possibly interpreted by the lower amount of APP, which can be dispersed more efficiently, leading to the blocking of segmental movements.

### 3.2. Limiting Oxygen Index (LOI) and UL-94 Standard Results

The LOI and UL-94 results of the flame retarded samples can be seen in Table 2.

By adding 1% P from RDP the LOI increased by 5% (*v/v*), and although the UL-94 classification remained HB, no horizontal burning rate could be measured. By further increase in P to 3% the LOI increased to 28% (*v/v*) however, even with 3% P from RDP only the HB UL-94 classification was reached. A further increase of the additive ratio was not possible due to deteriorating mechanical properties of the polymer: in order to introduce 4% P almost 40% of liquid RDP is necessary (due to its relatively low P-content), however, at this high ratio the too-large plasticizing effect of RDP would be unacceptable.

As for APP, with 1% P the LOI increased by 7% (*v/v*) to 27% (*v/v*), while in case of 3% P LOI of 31% (*v/v*) was reached. But similarly to RDP the UL-94 classification remained HB also in case of APP. A further increase of APP content was not preferred as the aggregation of the solid particles would be more pronounced, leading to increased viscosity and decreased mechanical properties (due to the introduced failure points).

Consequently, when applied alone, both the RDP and APP-containing formulations showed increased LOI values but their UL-94 ratings remained HB. Phosphorus content of 3% is generally sufficient to reach appropriate flame retardancy according to earlier experiences [25], thus, mixed flame retardant (FR) formulations with combined RDP and APP, have been also prepared. When 1% of phosphorus was introduced from RDP and 2 wt % from APP, the UL-94 rating remained HB, but inverting the ratio, and balancing it between the two additives led to a self-extinguishing V-0 rating with LOI values of 33%–34% (*v/v*). Comparing the HB-rated RDP (1% P) + APP (2% P) sample with the APP (2% P) sample, it can be concluded that by adding 1% P from RDP, the LOI deteriorated by 1% (*v/v*), probably due to lower degradation temperature of RDP (compared to APP, see the thermogravimetric analysis (TGA) results in Section 3.4.) in addition to the elevated O_2_-concentration during the LOI test. On the other hand, at ambient O_2_-concentration during the UL-94 test, due to the addition of 1% P from RDP, the sample failed the vertical ignition test only during the second ignition, similarly to samples containing 3% P from RDP or APP alone. In 3% P-containing mixed FR formulations at least 1.5% P from RDP and 1% P from APP, respectively, was necessary to reach the V-0 rate (in addition to the complementing FR added to reach 3% P-content). As, according to the literature, APP acts in the solid phase [7,8], while RDP acts mainly in the gas phase [8,11,12,13,14,15,16], it can be presumed that a balanced solid and gas phase FR action is responsible for the experienced synergistic results.

### 3.3. Mass Loss Calorimetry Results

**Figure 2:**
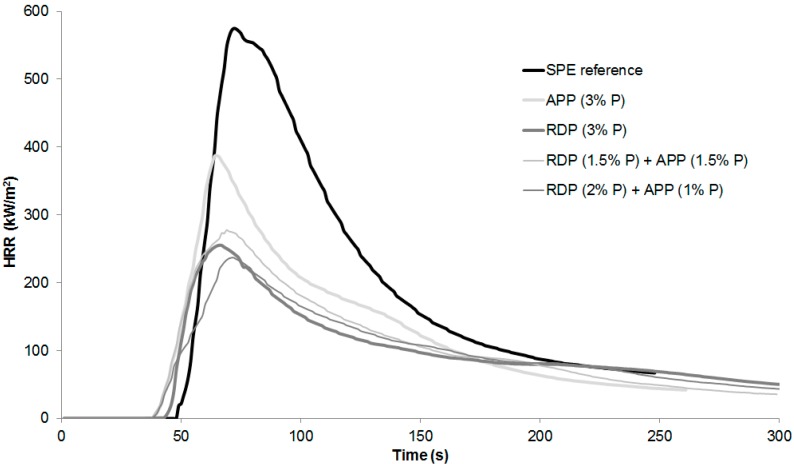
Heat release rate of reference and flame-retarded SPE samples.

**Figure 3:**
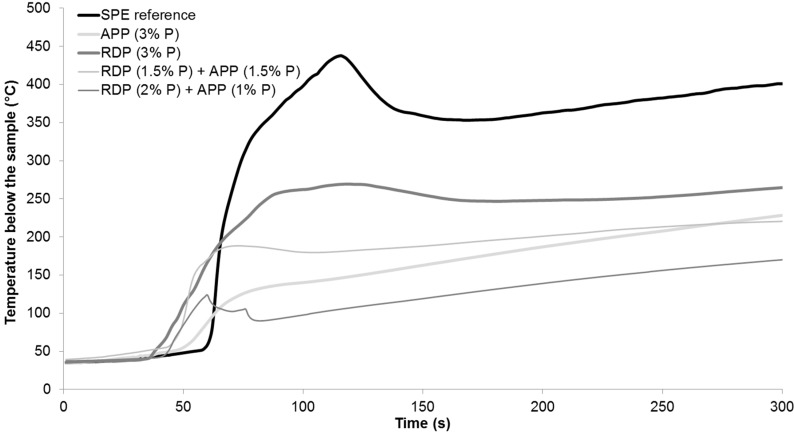
Average temperatures below the reference and flame-retarded SPE samples during mass loss calorimetry tests.

Specimens were prepared for mass loss calorimetry tests using the SPE reference, RDP (3% P), APP (3% P), and 3% P-containing mixed formulations reaching V-0 UL-94. The heat release rate curves can be seen in Figure 2, while the average temperature measured below the samples during the measurement is displayed in Figure 3. Numerical data obtained from mass loss calorimetry results are summarized in Table 3, best performances among the samples are highlighted with bold letters.

According to the results in case of combined FR samples the ignition occurred earlier, however the time to peak heat release rate (pHRR) increased compared to RDP (3% P) and APP (3% P) samples. From all formulations the RDP (2% P) + APP (1% P) sample had the lowest pHRR, FIGRA (fire growth rate), EHC (average effective heat of combustion), and MARHE (maximum of average rate of heat emission) so, similarly to the conclusions of the LOI and UL-94 test, this formulation can be considered as having the best overall performance. As for the average temperature measured below the samples in mass loss calorimeter, it can be concluded that, due to the earlier ignition in the case of FR samples, the temperature also started to rise earlier than in the case of SPE. After the ignition of the SPE sample the temperature measured below it increased suddenly, reaching a maximum of 437 °C. In flame-retarded samples, on the other hand, the formed charred layer acted as a heat barrier; therefore, the temperatures measured below the samples were lower than in case of SPE. After the ignition of SPE, among the FR samples lowest temperature was detected in case of combined RDP (2% P) + APP (1% P) specimen, which had a temperature of 170 °C at the end of the test, compared to 400 °C measured in case of SPE reference.

In order to explain the results of fire tests, the mode of action of flame retardants should be taken into account. The general opinion is that the ammonium polyphosphate acts in the solid phase as charring agent [7,8], while organophosphates act rather as radical scavengers in the gas phase [5,6,8,11,12,13,14,15,16]. Presumably, with the application of the combined FR formulation, a balanced solid and gas-phase mechanism was reached. To confirm this hypothesis, thermogravimetric analysis was carried out; furthermore, the composition of gas phase (formed during pyrolysis) and solid phase and strength of the charred residue were investigated as well.

### 3.4. Thermogravimetric Analysis

**Figure 4:**
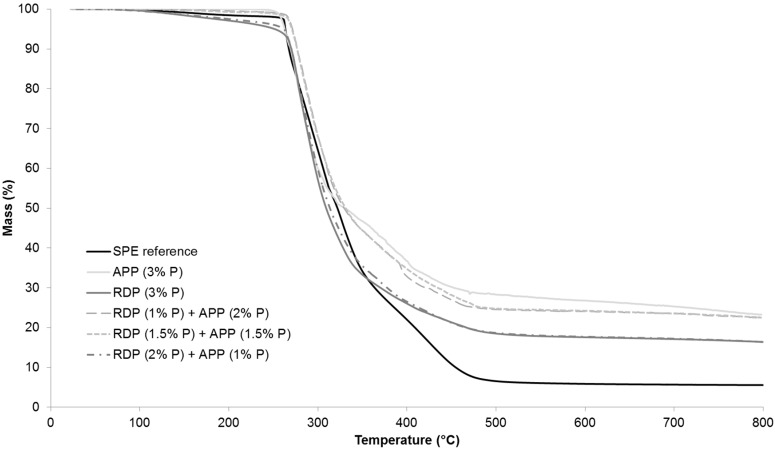
Full temperature range thermogravimetric analysis (TGA) curves of the reference and 3% P-containing SPE samples.

**Figure 5:**
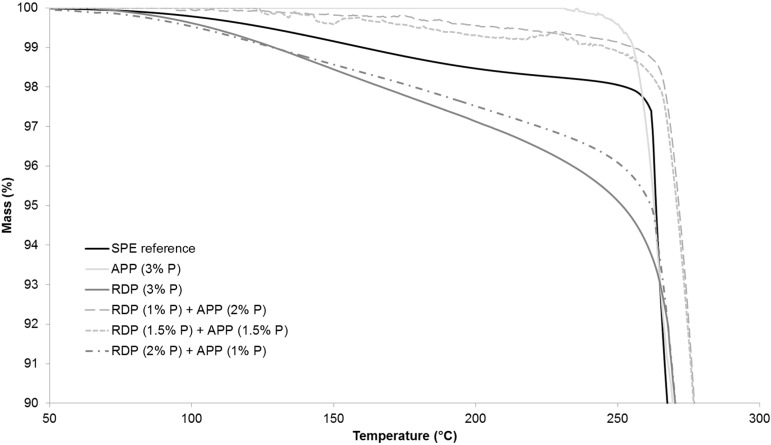
TGA curves of the reference and 3% P-containing SPE samples in the range of 50–300 °C.

The thermal stability of the reference and flame retarded SPE samples were examined by thermogravimetric analysis. Table 4 shows the temperature at 5% and 50% mass loss (*T*_-5%_; *T*_-50%_), the maximum mass loss rate (dTG_max_), the temperature belonging to this value (*T*_dTGmax_) and the char yield at the end of the TGA test (at 800 °C). The TGA curves in full temperature range from 25–800 °C are displayed in Figure 4, while Figure 5 shows the TGA curves from 50–300 °C in order to highlight the differences at the beginning of thermal degradation.

As it can be seen from Table 4, by increasing the amount of P introduced by RDP to 2% and 3%, the temperature belonging to 5% mass loss was gradually shifted to lower temperatures. The reason for this is that organic phosphorus flame retardants usually act during the early degradation step in the gas phase (for gas phase analysis results see Section 3.5). The temperature belonging to the 50% mass loss was around 15 °C less than in case of the reference sample. It was independent from the P content, as well as the maximum rate of mass loss. The char yield at 800 °C increased almost by 10% by introducing only 1% P, however, a further increase of P content did not result in significantly further improvement.

In the case of APP at 1% and 2% P content the temperature belonging to 5% mass loss increased compared to the reference, while the sample with 3% P showed similar value as the reference. The temperature belonging to 50% mass loss increased gradually with increasing the P content, while the maximum mass loss rate decreased. In the 3% P-containing sample the maximum mass loss rate was 60% lower than in case of the reference. The char yield at 800 °C was the same in the APP-containing sample with 1% P and in the RDP-containing sample with 3% P, confirming the solid phase mechanism of APP. The char yield gradually increases when the P content of APP origin is increased: at 3% P content, 25% of the initial sample mass remained as char at 800 °C.

As for the samples containing both RDP and APP, formulations containing 1% and 1.5% P of RDP origin showed very similar thermal behaviour (Figure 5), (the sample with 1% P and 1.5% P from RDP had UL-94 classification of HB and V-0, respectively (as shown in Section 3.2)). By increasing the amount of RDP in combined samples the thermal degradation started at a lower temperature and the temperature belonging to 50% mass loss decreased. On the other hand, by increasing the amount of APP the maximum mass loss rate decreased and the char yield increased. The different phase mechanisms of the two flame retardants could by clearly identified from the TGA results.

### 3.5. Investigation of Gas and Solid Phase Flame Retardancy Mechanisms by Infrared Spectrometry

**Figure 6:**
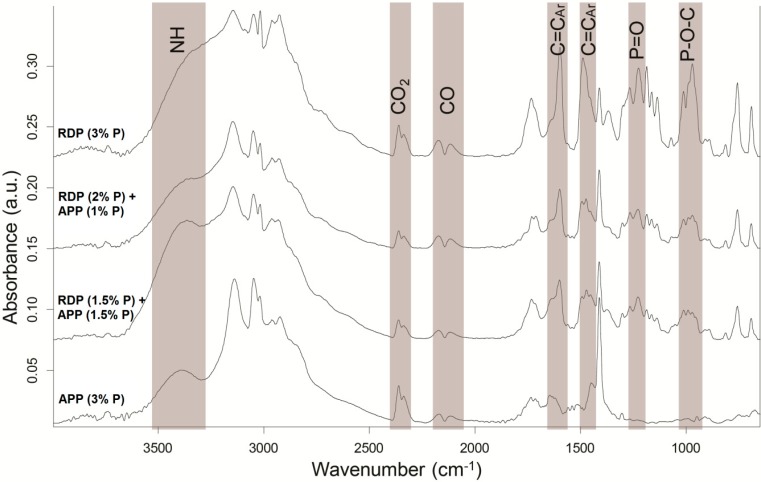
Laser pyrolysis–Fourier transform infrared (LP-FTIR) spectra of the gas phase degradation products from 3% P-containing samples.

Gas phase flame retardancy mechanism was examined by a coupled LP-FTIR method [19] in the case of four samples containing 3% P: samples containing only APP and only RDP, as well as the two samples reaching V-0 UL-94 classification (RDP (1.5% P) + APP (1.5% P) and RDP (2% P) + APP (1% P)) (Figure 6).

Based on Figure 6, clear differences could be identified in the gas phase spectra of different formulations. Whereas the vibrations belonging to P=O and P–O–C bonds appear as sharp peaks (in the range of 1290–1190 cm^−1^ and 1050 to 950 cm^−1^, respectively) in the case of the samples containing RDP, the sample containing only APP showed no peaks in these intervals. For all samples CO_2_ (2400–2300 cm^−1^) and CO (2200–2080 cm^−1^) peaks were observed in the gas phase, as well as aromatic C=C vibrations (1600 and 1490 cm^−1^, respectively). The intensity of the latter ones increased by increasing the RDP content. Ammonia formation could not be detected in the APP-containing samples, however, it could be seen that increasing the APP content the wide peak, characteristic for N–H vibrations (3400 cm^−1^), became more and more separated from the set of C–H vibrations (3200–2800 cm^−1^). Based on these results, no gas-phase effect could be detected in the case of the “pure” APP-containing sample, while with increasing RDP content, the amount of P species increased among the gas-phase degradation products.

**Figure 7:**
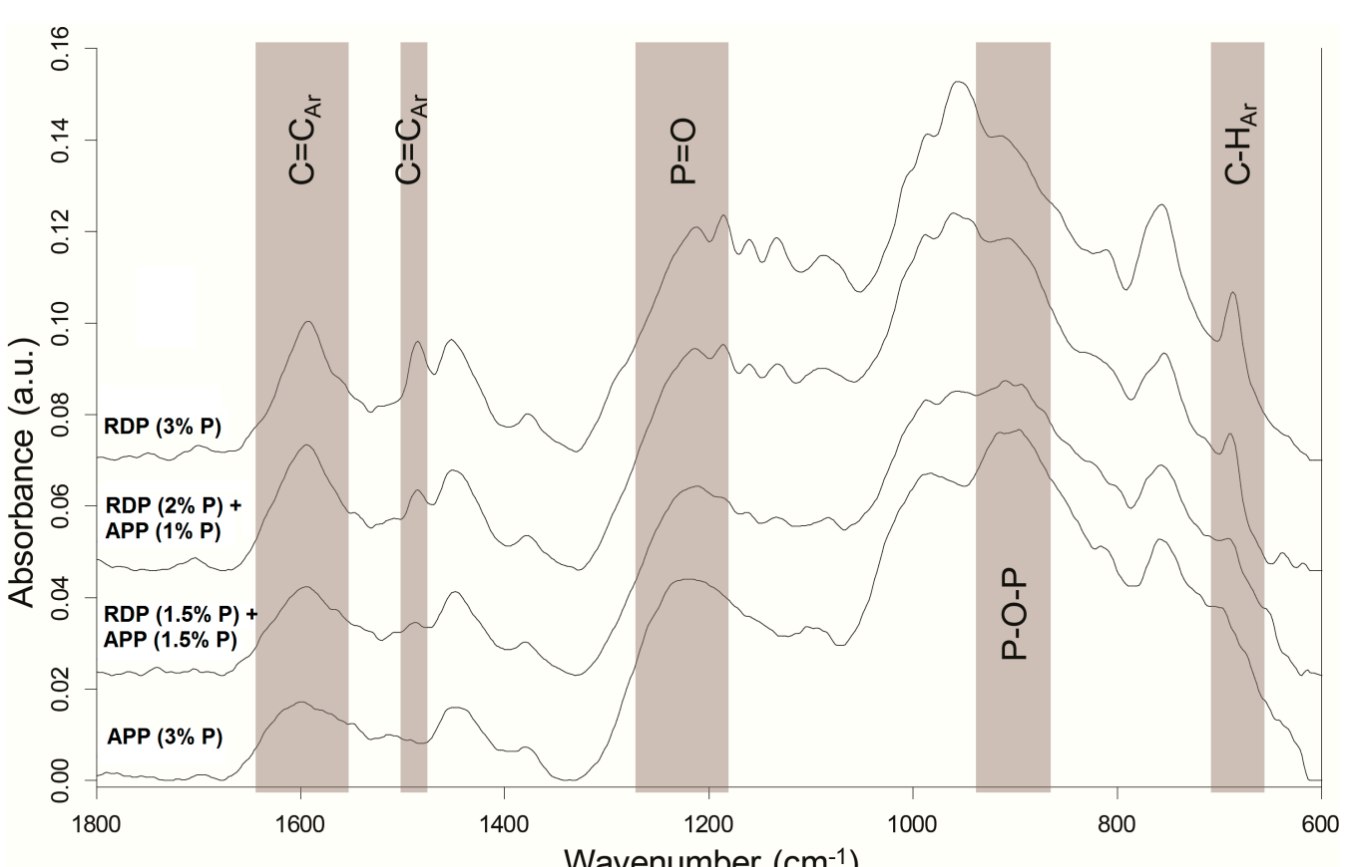
Attenuated total reflection-infrared spectrometry (ATR-IR) spectra of the charred residues from 3% P-containing samples.

Solid residues collected after 50 kW/m^2^ heat treatment in mass loss calorimeter were subjected to ATR-IR analysis (Figure 7).

Although in APP-containing samples the amount of charred residues is significantly higher, in their residues the peaks characteristic for aromatic C=C (1600 and 1480 cm^−1^) and C–H (690 cm^−1^) vibrations have lower intensity than in case of RDP-containing samples. The P content of RDP is only 10.8%, consequently to reach the same P content much more additive is needed than in the case of APP (containing 31%–32% P), and approximately 90% of the RDP’s remaining mass is present in the form of phenol and resorcinol, increasing the aromatic content of the solid phase residue.

On the other hand the intensity of the P–O–P (910 cm^−1^) and P=O (1215 cm^−1^) bonds in the FTIR spectrum of the charred residue is higher in the case of the APP (3% P) sample, and decreases with decreasing amounts of APP, which indicates the dominance of the solid-phase mechanism of the APP.

### 3.6. Char Strength Measurements

**Figure 8:**
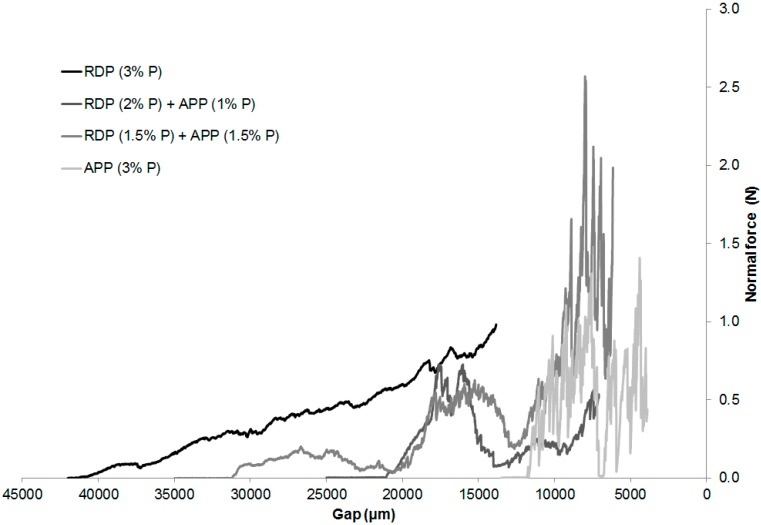
Compression strength results of SPE and flame-retarded SPE chars.

The mechanical resistance of the chars obtained after combustion in the mass loss calorimeter (set to 50 kW/m^2^ heat flux) was examined through compression tests carried out in a rheometer [20,21,22,23]. The average height of the charred residues is summarized in Table 5. The normal force transduced by the charred layer in the function of the distance between the two plates of the rheometer can be seen in Figure 8. After breaking the charred structure the normal force increases significantly because of the compression of the charred layer. The scattering of the normal force correlates with the diameter of the formed bubbles in the char: small, uniform fluctuation refers to small bubble diameter and uniform, flexible char; while a sudden decrease in the normal force proves the presence of bubbles with large diameter, which causes the char to have an uneven, rigid structure.

The height of the char was the biggest in case of SPE RDP (3% P) sample (42 mm). By decreasing the gap between the plates, the normal force transduced by the char increased almost linearly. Consequently, it can be assumed that the RDP entering the gas phase was capable to foam the upper layer of the polymer, forming a sponge-like, elastic, microporous char.

The sample flame retarded only with APP formed the smallest amount of char, which could be cracked with a minimal force (even by touching it by fingertips). In this case no monotone increase was detected, however, significant scattering of the normal force was typical, which means that an uneven, rigid structure was formed. As APP acts mainly in the solid phase, and the gas formation was less significant than in the case of RDP, only the slight upper layer of the polymer was foamed, which was easily destructed in the weaker points by the applied pressure.

Increasing tendency of normal force was detected in case of combined flame retardant system similarly to the sample containing only RDP. However, due to the APP induced rigid char, scattering of the normal force was also detected similarly to the sample containing only APP. From the fire retardancy point of view neither the too rigid, nor the too elastic char structure is favourable. The behaviour of the char formed in the case of the combined flame retardant compositions lies between the two extremes, which provided adequate protection.

## 4. Conclusions

In this work systematic study was carried out on the flame retardancy mechanism of combinations of solid and gas phase flame retardants. Fire retardancy of sorbitol polyglycidyl ether (SPE) bioepoxy resin was examined using (liquid) resorcinol bis(diphenyl phosphate) (RDP) of, mainly, gas phase action and (solid) ammonium polyphosphate (APP) acting in solid phase, and their combinations.

Regarding the effect of FRs on the *T*_g_, the plasticizing effect of RDP having low P-content was more pronounced, while in mixed FR formulations the *T*_g_ decrease was even less than in case of APP (their *T*_g_ decreased only by 10 °C).

As for the fire retardancy results, the formulations containing RDP or APP alone showed increased LOI values; nevertheless, self-extinction was only reached when the two additives were combined. The RDP (2% P) + APP (1% P) sample had the lowest pHRR, FIGRA, EHC, and MARHE values, so this formulation can be considered as having best overall fire performance.

The suspected balanced gas and solid-phase mechanism was first investigated by TGA analysis. In samples containing both RDP and APP the thermal degradation started at lower temperature when the amount of RDP was increased and the temperature belonging to 50% mass loss decreased. By increasing the amount of APP the maximum mass loss rate decreased and the char yield increased. The different phase mechanism of the two flame retardants could by clearly identified from the TGA results. LP-FTIR measurements indicated no gas-phase effect in the case of sample containing only APP, while increasing the RDP content lead to increased amount of P species among the gas-phase degradation products. RDP increased the aromatic content of the residue according to its ATR-IR spectra. On the other hand the dominance of the solid-phase mechanism in case of APP-containing samples was confirmed by the increasing intensity of the P–O–P (910 cm^−1^) and P=O (1215 cm^−1^) bonds in the FTIR spectrum as the APP-content increased. From the fire retardancy point of view neither the too rigid, nor the too elastic char structure is favourable. The char formed in the case of the combined flame retardant system laid between the two extremes, providing adequate protection.

Based on fire performance and proven balanced gas and solid-phase mechanism, a synergistic effect has been demonstrated between the solid, intumescent-type flame retardant (ammonium polyphosphate, APP) and the liquid resorcinol bis(diphenyl phosphate) (RDP) (which acts mainly in the gas phase as a radical scavenger).

## Figures and Tables

**Table 1:** Effect of the additive flame retardants on the glass transition temperature (*T*_g_) of SPE.

Flame retardant composition	*T*_g_ (°C)
SPE reference	124
RDP (1% P)	114
RDP (2% P)	108
RDP (3% P)	95
APP (1% P)	110
APP (2% P)	110
APP (3% P)	110
RDP (1% P) + APP (2% P)	114
RDP (1.5% P) + APP (1.5% P)	114
RDP (2% P) + APP (1% P)	114

**Table 2:** Limiting oxygen index (LOI) and UL-94 results of the flame-retarded SPE samples.

Flame retardant composition	LOI (%, *v/v*)	UL-94 (burning rate)
SPE reference	20	HB (20 mm/min)
RDP (1% P)	25	HB (vertical first ignition)
RDP (2% P)	27	HB (vertical first ignition)
RDP (3% P)	28	HB (vertical second ignition)
APP (1% P)	27	HB (vertical first ignition)
APP (2% P)	30	HB (vertical first ignition)
APP (3% P)	31	HB (vertical second ignition)
RDP (1% P) + APP (2% P)	29	HB (vertical second ignition)
RDP (1.5% P) + APP (1.5% P)	33	V-0
RDP (2% P) + APP (1% P)	34	V-0

**Table 3:** Mass loss type cone calorimetry results of reference and flame-retarded SPE samples.

Sample	TTI (s)	pHRR (kW/m^2^)	Time of pHRR (s)	FIGRA (kW/m^2^s)	THR (mJ/m^2^)	EHC (MJ/kg)	MARHE (kW/m^2^)	Residue (wt %)
SPE reference	45	575	72	8.0	43.2	18.7	233.1	5
RDP (3% P)	43	255	66	3.9	24.5	13.3	111.7	28
APP (3% P)	38	387	65	6.0	30.1	14.7	156.6	17
RDP (1.5% P) + APP (1.5% P)	31	278	69	4.0	26.7	13.7	129.8	20
RDP (2% P) + APP (1% P)	35	237	72	3.3	24.7	12.0	110.8	19

TTI: time to ignition; pHRR: peak of heat release rate; FIGRA: fire growth rate; THR: total heat released; EHC: average effective heat of combustion; MARHE: maximum of average rate of heat emission); average standard deviation of the measured mass loss calorimeter values: TTI: ± 3, pHRR: ± 30, time of pHRR: ± 5, residue: ± 2.

**Table 4:** *T*_-__5%_; *T*_-__50%_; dTG_max_; *T*_dTGmax_ and char yield values of flame-retarded SPE samples.

Flame retardant composition	*T*_-5%_ (°C)	*T*_-50%_ (°C)	dTG_max_ (%/°C)	*T*_dTGmax_ (°C)	Char yield (%)
SPE reference	263.5	322.1	−1.5	263.2	5.6
RDP (1% P)	264.2	309.1	−1.2	274.6	13.4
RDP (2% P)	258.9	309.3	−1.2	275.4	15.5
RDP (3% P)	250.7	309.4	−1.5	277.3	16.6
APP (1% P)	276.3	309.5	−1.8	283.9	16.4
APP (2% P)	272.2	319.2	−1.4	277.9	19.8
APP (3% P)	262.7	330.2	−0.6	275.7	23.2
RDP (1% P) + APP (2% P)	271.6	329.5	−1.0	286.0	22.5
RDP (1.5% P) + APP (1.5% P)	270.6	331.2	−1.0	278.9	22.5
RDP (2% P) + APP (1% P)	261.6	313.6	−1.2	273.5	16.6

*T*_-5%_: temperature at 5% mass loss; *T*_-50%_: temperature at 50% mass loss; dTG_max_: maximum mass loss rate; *T*_dTGmax_: the temperature belonging to maximum mass loss rate.

**Table 5:** Average heights of the charred residues.

Sample	Average height of three samples (mm)
RDP (3% P)	42 ± 3
APP (3% P)	11 ± 1
RDP (1.5% P) + APP (1.5% P)	31 ± 2
RDP (2% P) + APP (1% P)	27 ± 2

## Abbreviations

SPE	Sorbitol polyglycidyl ether
RDP	Resorcinol bis(diphenyl phosphate)
APP	Ammonium polyphosphate
LOI	Limiting oxygen index
FTIR	Fourier transform infrared spectrometry
ATR-IR	Attenuated total reflection-infrared spectrometry
FR	Flame retardant
TGA	Thermogravimetric analysis
DGEBA	Diglycidyl ether of bisphenol-A
EP	Epoxy resin
DSC	Differential scanning calorimetry
LP-FTIR	Laser pyrolysis-FTIR coupled method
DTGS	Deuterated triglycine sulfate
pHRR	Peak of heat release rate
FIGRA	Fire growth rate
EHC	Average effective heat of combustion
MARHE	Maximum of average rate of heat emission
TTI	Time to ignition
THR	Total heat released
